# Fatty acid binding protein 3 (*fabp3*) is associated with insulin, lipids and cardiovascular phenotypes of the metabolic syndrome through epigenetic modifications in a northern european family population

**DOI:** 10.1186/1755-8794-6-9

**Published:** 2013-03-19

**Authors:** Yi Zhang, Jack W Kent, Adam Lee, Diana Cerjak, Omar Ali, Robert Diasio, Michael Olivier, John Blangero, Melanie A Carless, Ahmed H Kissebah

**Affiliations:** 1 TOPS Obesity and Metabolic Research Center, Department of Medicine, Medical College of Wisconsin, Milwaukee, Wisconsin 53226, USA; 2 Human and Molecular Genetics Center, Medical College of Wisconsin, Milwaukee, Wisconsin 53226, USA; 3 Department of Genetics, Texas Biomedical Research Institute, San Antonio, Texas 78245, USA; 4 Molecular Pharmacology and Experimental Therapeutics, Mayo Clinic, Rochester, Minnesota 55905, USA; 5 Department of Pediatrics, Medical College of Wisconsin, Milwaukee, Wisconsin 53226, USA; 6 Department of Physiology, Medical College of Wisconsin, Milwaukee, Wisconsin 53226, USA; 7 Biotechnology and Bioengineering Center, Medical College of Wisconsin, Milwaukee, Wisconsin 53226, USA

**Keywords:** Epigenetic regulation, Metabolic syndrome, Fatty acid binding proteins, Family studies, Association studies

## Abstract

**Background:**

Fatty acid-binding proteins (FABPs) play regulatory roles at the nexus of lipid metabolism and signaling. Dyslipidemia in clinical manifestation frequently co-occurs with obesity, insulin resistance and hypertension in the Metabolic Syndrome (MetS). Animal studies have suggested FABPs play regulatory roles in expressing MetS phenotypes. In our family cohort of Northern European descent, transcript levels in peripheral white blood cells (PWBCs) of a key FABPs, FABP3, is correlated with the MetS leading components. However, evidence supporting the functions of FABPs in humans using genetic approaches has been scarce, suggesting FABPs may be under epigenetic regulation. The objective of this study was to test the hypothesis that CpG methylation status of a key regulator of lipid homeostasis, *FABP3*, is a quantitative trait associated with status of MetS phenotypes in humans.

**Methods:**

We used a mass-spec based quantitative method, EpiTYPER*®*, to profile a CpG island that extends from the promoter to the first exon of the FABP3 gene in our family-based cohort of Northern European descent (n=517). We then conducted statistical analysis of the quantitative relationship of CpG methylation and MetS measures following the variance-component association model. Heritability of each methylation and the effect of age and sex on CpG methylation were also assessed in our families.

**Results:**

We find that methylation levels of individual CpG units and the regional average are heritable and significantly influenced by age and sex. Regional methylation was strongly associated with plasma total cholesterol (p=0.00028) and suggestively associated with LDL-cholesterol (p=0.00495). Methylation at individual units was significantly associated with insulin sensitivity, lipid particle sizing and diastolic blood pressure (p<0.0028, corrected for multiple testing for each trait). Peripheral white blood cell (PWBC) expression of FABP3 in a separate group of subjects (n=128) negatively correlated with adverse profiles of metabolism (β_WHR_ = −0.72; β_LDL-c_ = −0.53) while positively correlated with plasma adiponectin (β=0.24). Further, we show that differential methylation of *FABP3* affects binding activity with nuclear proteins from heart tissue. This region that we found under methylation regulation overlaps with a region actively modified by histone codes in the newly available ENCODE data.

**Conclusions:**

Our findings suggest that DNA methylation of *FABP3* strongly influences MetS, and this may have important implications for cardiovascular disease.

## Background

Lipids serve the body not only as major metabolic fuels but also as membrane signaling transducers and modulators of nuclear transcription factors [1]. Lipid homeostasis is therefore under sophisticated regulation and is well connected with other pathways of metabolism. An unbalanced lipid state is often clustered with obesity, insulin resistance and hypertension, which as a whole is a common metabolic phenomenon called the Metabolic Syndrome (MetS) [2]. Long-chain fatty acids (LCFAs) are a major type of lipid but free LCFAs are insoluble in the cytosol. Their movement within the cell requires facilitation of protein chaperones, the fatty acid binding proteins (FABPs), a family of soluble cytosolic small (14–15 kDa) polypeptides that bind with LCFAs [3]. There is increasing evidence that different types of FABPs are important contributors to the development of the MetS [4,5]. Furthermore, serum levels of the heart type fatty acid binding protein (H-FABP), has been shown to be associated with MetS in patients [6].

H-FABP, encoded by the *FABP3* gene, is widely distributed with highest levels found in the heart and smaller amounts present in slow skeletal muscle, testes, fast skeletal muscle, brain, kidney, lung, adrenal gland [7,8], and in lymphocytes [9]. Studies in animal models have shown that H-FABP helps maintain a balanced energy supply to heart [10] and other body parts [11], regulates intramuscular fat content and adipose tissue development [12], improves insulin sensitivity [13], and regulates dopamine D2 receptor function in the brain [14]. Such evidence would suggest a potential role for H-FABP in MetS in humans. Indeed, serum levels of H-FABP have been shown to be correlated with body mass index (BMI), weakly correlated with hypertension [15] and elevated in pre-diabetic patients [16], and MetS patients [6]. Given that protein levels of H-FABP fluctuate in parallel with mRNA levels, regulation is likely to be at the transcriptional level [17]. In our cohort of extended Northern European families, the Metabolic Risk Complications of Obesity Genes (MRC-OB) cohort, transcript levels of FABP3 from peripheral white blood cells (PWBCs) has been found to be suggestively correlated (p<0.1) with the MetS key axis phenotype defined by the two leading components waist circumference (WC) and homeostasis model assessment (HOMA) (unpublished data) [2,18-20]. Given the fact that DNA sequence variation within *FABP3* has rarely been found to be associated with human disease states [21,22], it is possible that *FABP3* transcription is regulated by epigenetic factors.

DNA CpG methylation is the most studied aspect of the epigenetic code. It is fundamental to the biology of the cell because newly added methyl groups can prevent the binding of transcription factors or attracts methyl-binding domain (MBD)-containing proteins that can recruit transcription suppressors such as histone deacetylases [23]. There is increasing evidence that regulatory activity at the level of DNA methylation plays an integral part in the causation of complex human diseases, including cancer, neuronal diseases, diabetes and obesity [24-28]. It is known that cells of different tissue types display distinct patterns in global CpG methylation [29]. In this study of roles of epigenetic variation in disposing MetS traits, we assayed DNA CpG methylation in white blood cells of peripheral blood based on the following reasons. First, obesity is considered a low-grad inflammation state [30] and macrophage accumulation in adipose tissue is an early event in this chronic inflammation [31,32]. Profiling CpG methylation in the tissue of peripheral blood thus could detect MetS-specific epigenetic changes. Second, obtaining genomic CpG methylation information using peripheral blood is minimally invasive, making it possible to assay in large number of our subjects. Third, there is evidence showing concordance in CpG methylation profiles between peripheral blood and other tissue types [29] suggesting peripheral blood can be a surrogate tissue source for epigenetic studies in humans. Recent efforts to identify epigenetic markers associated with obesity and type 2 diabetes in humans, including those done in peripheral blood, revealed a few candidate genes whose transcription is under epigenetic regulation through modifications of the methylation of CpG sites within or near promoter regions [33-36]. Studies that carefully examine the characteristics of CpG methylation in family settings in relation to complex diseases like obesity and metabolic syndrome are, however, very scarce. We report here our findings on the methylation profiles of a CpG island near *FABP3*, a gene whose product H-FABP is of high biological importance in energy and metabolic homeostasis.

A recent pilot study examining the effects of DNA methylation on cardiovascular-related phenotypes within the San Antonio Family Heart Study (SAFHS) [37] showed suggestive correlation between methylation levels at a CpG site within exon 1 of *FABP3* and several metabolic syndrome (MetS)-related phenotypes, including HDL-cholesterol (p=0.0017) and fasting insulin (p=0.0048) (unpublished data; p-values not corrected for multiple testing). We therefore conducted a study to test whether the proposed function of *FABP3* in MetS is under epigenetic regulation using families of our MRC-OB cohort shown to be highly informative for finding genetic elements important for MetS-associated lipid pathways [38,39]. To finely dissect the relationship between quantitative methylation and the clinical outcomes as well as the biological precursor phenotypes expressed in MetS, we examined our extensively phenotyped cohort and identified several CpG sites that were associated with lipids, insulin and blood pressure measures. To our knowledge, this is the first study describing a role for epigenetic regulation of *FABP3* in metabolic syndrome traits.

## Methods

### Subjects and phenotypes

The study cohort consists of 517 individuals representing 40 extended nuclear families. Details of recruitment and phenotyping procedures have been described previously [40]. Briefly, each family was recruited through an obese proband (BMI ≥ 30) with the minimal requirement of the availability of one obese sibling and one never-obese (BMI ≤ 27) sibling and availability of at least one, preferably both, of the parents. Clinical phenotypes included weight, height, BMI, waist circumference (WC), hip circumference (HC), waist to hip ratio (WHR), fasting glucose (FG), fasting insulin (FI), insulin to glucose ratio (IGR), homeostasis model assessment (HOMA), plasma triglycerides (TG), total cholesterol (TC), low density lipoprotein- cholesterol (LDL-c) and calculated LDL-c levels (cal. LDL-c), high density lipoprotein-cholesterol (HDL-c), systolic and diastolic blood pressure (sBP and dBP) and pulse. Biological phenotypes were determined according to standard published procedures, and included: measurement of total fat mass in kilograms and percentage (Fatkg and Fatpct), and lean mass in kilograms and percentage (Leankg and Leanpct) by Dual-emission X-ray absorptiometry (DEXA) [41]; total abdominal, visceral and subcutaneous fat sizes (TAF, VF and SubQF) as measured by computed tomography (CT/MRI) scans of an average of three sections at the fourth lumbar spine [42]; resting energy expenditure (REE) and respiratory quotient (RQ) as measured in resting subjects using a Deltatrac Indirect Calorimeter (Sensor Medics, VIASYS Healthcare, Conshohocken,PA) after a 10 hr fast; insulin/glucose responsiveness indices of insulin sensitivity (SI), glucose effectiveness (SG), acute insulin response to glucose (AIRG) and disposition index (DI) by Minimal Model [43]; lipids/lipoprotein sizing [HDL median diameter (HMED), LDL-cholesterol median diameter (LMEDn), LDL-cholesterol dominant peak diameter (LDLppd) and apoB-containing non-HDL median diameter (BMED) which includes VLDL, ILDL, LPα and LDL] as measured by polyacrylamide gradient gel electrophoresis [44]; circulating levels of adiponectin and leptin measured by a double antibody equilibrium radioimmunoassay (RIA) (Millipore Corporation, Billerica, MA); and TNF-alpha, interleukin-1beta (IL-1β) and interleukin-6 (IL-6)] levels that were measured as previously described [45]. Informed consent was obtained from the participating subjects. All study procedures for all participants were approved by the Institutional Review Boards of the Medical College of Wisconsin (HRRC#013-00).

### Quantitative methylation analysis using EpiTYPER®

Methylation analysis was performed as described previously by Lee *et al.*[46]. Approximately 500 ng of genomic DNA isolated from PWBCs was bisulfite modified (BSM) using the EZ-96 DNA Methylation Kit following the manufacturer’s protocol (Zymo Research; Irvine, CA). Methylated and unmethylated control DNA (Zymo) were also bisulfite modified to confirm complete bisulfite modification and for quality control.

Three PCR primer regions were designed to cover the entire region of interest within the promoter and first exon region of *FABP3* (for details of their sequences and chromosomal positions, see Additional file 1: Table S1). *EpiTYPER®* CpG methylation profiling was done per manufacturer instruction, with modifications as described here. BSM DNA was PCR amplified using T7-promoter tagged primers specific for single-stranded, bisulfite-modified DNA. PCR amplification of each amplicon was performed in a 6 μl reaction volume (1x PCR Buffer, 0.75 mM MgCl_2_, dNTPs (2.5 mM each), 0.20 mM each primer, 0.05 μl HotStar Taq DNA polymerase (Qiagen; Valencia, CA) & 2 μl bisulfite-modified DNA). To ensure accurate DNA methylation detection, all bisulfite-modified samples were PCR amplified in triplet along with five methylation controls (obtained from the Zymo Control Set). These included 100%, 75%, 50%, 25% and 0% methylated bisulfite-converted DNA, obtained from appropriate combination of the methylated and unmethylated control DNA samples. Unincorporated dNTPs from the PCR reaction were neutralized using shrimp alkaline phosphatase (SAP), and double-stranded cRNA was created from the template by *in vitro* transcription. The cRNA was base-specifically cleaved with RNase A, generating fragments (CpG units) containing any number of CpG sites. The cleavage products were then spotted onto silicon chips (SpectroCHIP II, Sequenom) using a nanodispenser. Adenine and guanine differ in mass by ~16 daltons; therefore, each CpG site that is methylated creates a 16 dalton change that is detectable by matrix assisted laser desorption ionization-time of flight mass spectrometry (MALDI-TOF MS) (MassARRAY® system, Sequenom).

**Figure 1:**
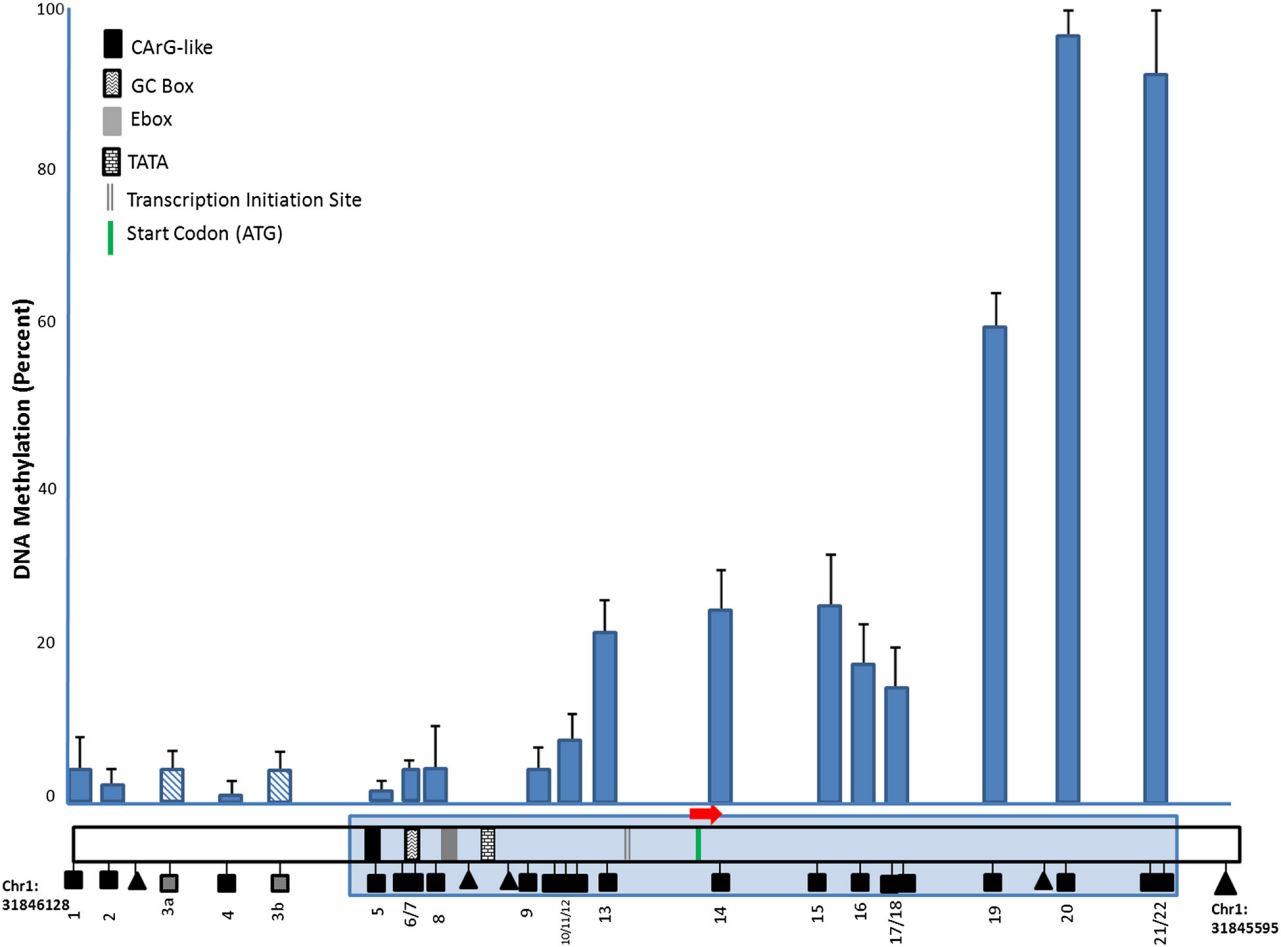
**CpG methylation profile of *FABP3 *promoter/1**^**st**^**exon region.** A total of 28 CpG sites were identified within or immediately upstream to the CpG island spanning the boundary between the promoter and the first exon of *FABP3* gene. Methylation values at each CpG loci measured in our MetS cohort (y-axis) were averaged and plotted against their chromosomal positions (x-axis). CpG methylation levels are depicted by bars with standard deviations shown in error bars. A graphical representation of the *FABP3* region under investigation is shown below the bar graph. Squares represent the CpG units that passed the quality control standard (see Materials and Methods). Triangles represent the CpG units that did not pass the quality control criteria and were therefore not analyzed further. The location of the identified CpG island is highlighted in blue. Transcription start site, position of the start codon and transcription elements that were previously identified in the promoter region (53) are also shown.

Methylation percentage for CpG sites within a sample of genomic DNA was calculated with the EpiTYPER software which generates predicted “CpG units”, which may contain one or more CpG sites, and pre-calculates their mass. The abundance of each fragment (signal/noise level in the spectrum) detected by the MassARRAY® system is indicative of the amount of DNA methylation in the interrogated sequence. The CpG sites whose methylation intensity cannot be unambiguously determined by the MassARRAY® technology were excluded from further analyses (see legend of Figure 1 for their locations). In some cases, CpG sites were within the same CpG unit and were unable to be differentiated; CpG sites 6/7, 10/11/12, 17/18 and 21/22 were therefore assessed as a single site. CpG sites 3a and 3b represent different cleavage fragments which possessed the same mass, and therefore could not be distinguished from each other. In this case, the methylation level is recorded as an average of the two fragments. We have rejected the entire methylation datasets of 13 subjects (2.5%) with calling rate lower than 95%. Of the remaining subjects, we excluded data points (4.8% of all measurements) that have standard deviation higher than 0.1.

### Transcriptional profiling

Genome-wide transcriptional profiles of a subset of the cohort (128 individuals from 55 families) were obtained as previously described [47] with modifications. Briefly, for each individual 2.5 ml blood was collected into a PAXgene® Blood RNA Tube (BD, Franklin Lakes, NJ) following an overnight fast. Total RNA was isolated from each tube using the PAXgene Blood RNA Kit (Qiagen, Valencia, CA) and anti-sense RNA (aRNA) was synthesized using the MessageAmp II-Biotin aRNA kit (Ambion, Austin, TX). A total of 1.5 μg aRNA was hybridized to Illumina HumanWG-6 version 2 or version 3 chips (Illumina, San Diego, CA) and expression detected on the Illumina® BeadArray™ 500GX Reader. Illumina GenomeStudio software (version 2010.3) was used for preliminary data analysis with standard background normalization.

### Electrophoretic mobility shift assay

Electrophoretic Mobility Shift Assay (EMSA) was performed on completely methylated and completely unmethylated DNA from the promoter region. First, PCR amplification of the target area was performed in a 20 μl volume [1x Buffer, dNTPs (0.8 mM each), 0.2 mM MgCl_2_, 2 mM each primer, 0.04 μl HotStar Taq DNA polymerase (Qiagen; Valencia, CA), and 4 μl CEPH (Centre d’Etude du Polymorphisme Humain) DNA] with an annealing temperature of 58°C for 40 cycles. Oligonucleotide primers were synthesized from IDT technologies (Coralville, IA) as follows; forward primer: 5^′^ TTGCTGTCCACTAGCTTCCA 3^′^ and reverse primer: 5^′^/biotin/ CCTCCTGGGTGAGCCCTA 3^′^. PCR products were size-selected using Invitrogen 2% Size Select™ E-Gel® and then purified with the PCR Purification Kit (Qiagen; Valencia, CA) per the manufacturers’ instructions.150 ng of the biotin-labeled DNA was treated with CpG Methylase (Zymo; Irvine, CA), by incubating at 30°C overnight, per manufacturer instruction. After an initial 2 hour incubation period, additional CpG Methylase (1 uL) was added, to drive the methylation to completion. The enzyme was inactivated at 65°C for 20min and the reaction was cleaned with the MinElute Reaction Cleanup Kit (Qiagen; Valencia, CA). To verify methylation, 50 ng each of methylated and unmethylated DNA was digested with HpaII (New England BioLabs; Ipswich, MA) at 37°C for 1 h. The enzyme was inactivated at 65°C for 20min. The reaction was cleaned with the MinElute Reaction Cleanup Kit (Qiagen; Valencia, CA) and checked by gel electrophoresis. Quantification of DNA was performed using Promega QuantiFluor dsDNA System (Madison, WI), as per the manufacturer’s multiwell plate instruction and plates read on a Thermo Scientific Varioskan Flash (Rockford, IL).

Electrophoresis mobility shift assays (EMSA) were performed using the LightShift EMSA kit (Thermo Scientific; Rockford, IL) with adaptations from previous studies [39,48]. For EMSA, methylated-biotin labeled DNA and unmethylated biotin-labeled DNA were used in separate reactions. Human heart nuclear lysate (left ventricle) was purchased from ProSci Incorporated (Poway, CA) and 3 μg was pre-incubated for 10 min on ice with 1 μg of poly(dI-dC), 10X Binding Buffer, 1% NP-40, MgCl_2_ and glycerol. Nuclear proteins were then incubated with 0.1 ng of methylated (or unmethylated for control) biotin-labeled DNA for 30 min on ice, analyzed on 6% Novex DNA retardation gels (Invitrogen; Carlsbad, CA) and electroblotted onto a positively charged nylon membrane (Invitrogen; Carlsbad, CA). Detection of protein/DNA complexes was achieved following incubation of the membrane with streptavidin-horseradish peroxidase and development with luminal substrate (Thermo Scientific; Rockford, IL). Light emission was captured on X-ray film.

### Statistical analysis

Descriptive statistics were computed using the R programming language [49]. Quantitative genetic analyses were performed using SOLAR [50]. Briefly: based on pedigree data, a kinship coefficient was computed for each pair of study subjects equal to the expected proportion of alleles shared identical by descent (= 0 for individuals without common ancestors in the pedigrees). Using this information, the phenotypic covariance between pairs of individuals was decomposed into expected variances due to additive genetics effects and residual random effects, respectively. The standardized additive genetic variance of a phenotype is its *heritability* (h^2^). All subsequent tests of the fixed effects of methylation status also included these random effects, so the non-independence of related subjects was properly accounted for. We also included in all models the fixed effects of sex, age, age^2^, sex by age interaction, and sex by age^2^ interaction.

To meet the distributional assumptions of the variance-decomposition tests, all phenotypes – the MetS traits as well as the percent-methylation measures – were normalized by inverse-Gaussian (rank-normal) transformation. All genetic models were tested by likelihood maximization in SOLAR. The test statistic for each hypothesis was equal to twice the difference in log_e_(likelihood) of the test model and its respective null. For fixed effects, this test statistic is distributed as chi-squared with degrees of freedom equal to the number of parameters under test; for h^2^ (which is tested on its lower boundary, h^2^= 0) the distribution is a 0.5:0.5 mixture of χ^2^_1_ and a point mass at zero [51].

Gene expression microarray data were available for a total of 128 samples (48,803 probes, Illumina Beadchips v3 arrays). The number of probe transcripts detectable at p≤0.05 by BeadStudio software was counted, a false discovery rate (FDR) was computed across all probes, and transcripts detectable at 5% FDR were retained. Expression levels were log_2_ transformed and inverse-quartile normalized. Transformed and normalized expression levels for probes that mapped to FABP3 were tested for correlation with phenotypes of interest in models that included the random effect of kinship.

Correction for multiple testing of the effects of the CpG units was based on the effective number of independent units given the potential correlation among units in the same genomic region. We used a method originally devised for association tests of SNP variants [52] as implemented in SOLAR [50]. The Bonferroni-Šidák significance threshold for multiple tests in the *FABP3* region was calculated using alpha = 0.05 (significant) and alpha = 0.1 (suggestive), and the effective number of tests.

## Results

### Clinical outcome and biological precursor phenotypes of the MetS study cohort

Table 1 shows the sex-specific means (±standard deviation, STD) of the 42 MetS phenotypes measured in 517 individuals of 40 extended families of Northern European descent. 58.9% of the cohort is female, the average age of the cohort is 43.6y and the average BMI is 32.937 kg/m^2^. Phenotypes have been grouped into 5 categories (body composition phenotypes, glucose and insulin responsiveness, lipid homeostasis, cardiovascular performance and adipokines/cytokines) that reflect major components of MetS. Table 1 also shows the estimated heritability of each MetS phenotype in our families. Heritability of these phenotypes varies from 0.12 for glucose effectiveness (SG) to 0.69 for height and HDL cholesterol.

**Table 1:** Phenotypic characteristics and heritability estimates of the MetS cohort

	**Trait**	**Mean ± STD (Male)**	**Mean ± STD (Female)**	**Heritability ± SE**
Body Composition	Weight, kg	97.73 ± 26.30	90.28 ± 25.92	0.40±0.10
	Height, cm	176.34 ± 6.97	163.46 ± 6.95	0.69±0.07
	BMI, kg/m_2_	31.45 ± 8.40	33.95 ± 10.19	0.43±0.09
	Waist circumference (WC), cm	102.12 ± 18.34	97.77 ± 20.16	0.33±0.09
	Hip circumference (HC), cm	109.44 ± 16.52	117.95 ± 20.40	0.43±0.10
	Waist to Hip ratio (WHR)	0.93 ± 0.08	0.83 ± 0.09	0.23±0.09
	Total Fat Mass (Fatkg), kg	28.49 ± 13.81	38.01 ± 14.68	0.41±0.11
	Total Fat Mass (Fatpct),%	29.78 ± 9.02	43.94 ± 7.79	0.39±0.10
	Total Lean Mass (Leankg), kg	62.53 ± 8.23	45.81 ± 8.44	0.55±0.09
	Total Lean Mass (Leanpct), %	74.29 ± 39.60	57.74 ± 28.07	0.38±0.10
	Subcutaneous Fat (SubQF), g	72.61 ± 44.52	93.54 ± 48.94	0.57±0.10
	Visceral Fat (VF), g	57.32 ± 30.54	42.86 ± 29.17	0.34±0.10
	Total Abdominal Fat (TAF), g	128.96 ± 66.28	136.82 ± 69.39	0.54±0.10
	Respiratory Quotient (RQ)	0.83 ± 0.09	0.83 ± 0.08	0.30±0.09
	Resting Energy Expenditure (REE), kcal/24 hrs	1931.18 ± 327.99	1620.70 ± 292.96	0.34±0.10
	REE/weight, kcal/24hrs/kg	20.17 ± 3.64	18.62 ± 3.21	0.37±0.11
	REE/Lean mass (REE/lean), kcal/24hrs/kg	30.37 ± 3.83	35.18 ± 4.28	0.36±0.11
Glucose and Insulin Responsiveness	Fasting Glucose (FG), mg/dl	88.53 ± 30.35	87.14 ± 30.83	0.51±0.09
	Fasting Insulin (FI),μU/ml	15.96 ± 12.90	19.24 ± 24.53	0.38±0.08
	Insulin/glucose (IGR)	0.27 ± 0.76	0.29 ± 0.93	0.35±0.08
	Homeostasis model assessment (HOMA)	3.52 ± 3.04	4.63 ± 9.92	0.38±0.08
	Insulin Sensitivity (SI), (× 10^-4^/min/μU/ml)	3.32 ± 2.94	2.86 ± 2.79	0.20±0.10
	Glucose Effectiveness (SG), min^-1^	0.02 ± 0.01	0.02 ± 0.02	0.12±0.10
	Acute Insulin Response to glucose (AIR_G_), μU/ml × 10min	537.09 ± 477.19	416.81 ± 338.89	0.12±0.08
	Disposition Index (DI), AUC (Insulin_0-10 min_) × SI	1367.12 ± 1188.95	1054.62 ± 996.43	0.25±0.09
Lipid Homeostasis	Triglycerides (TG), mg/dl	135.40 ± 208.06	106.45 ± 64.89	0.52±0.11
	Total cholesterol (TC), mg/dl	193.08 ± 45.75	189.06 ± 40.03	0.29±0.08
	LDL-cholesterol (LDL-c), mg/dl	131.44 ± 40.55	126.95 ± 36.61	0.33±0.09
	Calculated LDL-C (cal. LDL-c), mg/dl	134.83 ± 55.40	126.53 ± 38.97	0.45±0.10
	HDL-cholesterol (HDL-c), mg/dl	38.24 ± 12.05	43.65 ± 18.63	0.69±0.08
	HMED, nm	8.83 ± 0.31	9.19 ± 0.50	0.40±0.10
	LMEDn, nm	27.03 ± 0.70	27.26 ± 0.97	0.46±0.11
	LDL_PPD, nm	27.17 ± 0.95	27.99 ± 1.30	0.61±0.10
	BMED, nm	27.67 ± 1.09	28.07 ± 1.28	0.42±0.10
Cardiovascular Performance	Systolic Blood Pressure (sBP), mmHg	128.69 ± 15.34	124.90 ± 19.07	0.22±0.09
	Diastolic Blood Pressure (dBP), mmHg	78.97 ± 12.18	75.68 ± 10.74	0.22±0.10
	Pulse, beats/min	68.59 ± 9.85	72.93 ± 21.31	0.15±0.08
Adipokines and Cytokines	Adiponectin, ng/ml	6.66 ± 3.06	9.47 ± 4.77	0.60±0.09
	Leptin, ng/ml	11.07 ± 10.90	25.34 ± 15.19	0.41±0.09
	TNF-alpha, pg/ml	4.26 ± 2.67	3.74 ± 2.53	0.38±0.09
	Interleukin-1 beta (IL-1β)	0.40 ± 0.95	0.46 ± 0.96	0.23±0.10
	Interleukin-6 (IL-6)	3.75 ± 8.53	5.42 ± 9.44	0.20±0.09

The mean +/− standard deviation is shown for each of the 42 MetS phenotypes. Heritability of MetS phenotypes estimated in these 40 extended families (n=517) is shown with standard errors (SE). For nomenclature of the 42 phenotypes, refer to Materials and Methods.

### CpG methylation profile of the promoter region of FABP3

The 5^′^ region immediately upstream of the transcription start site of *FABP3* has been shown to be sufficient to drive the expression of this gene in a mouse model [53]. We examined the genomic sequence adjacent to the transcriptional start site (TSS) and identified a CpG island that spans the region upstream of the TSS (−132 bp) to the first exon (+185 bp). We included in our analysis of CpG sites that were located immediately upstream to the beginning of the island. This 533 bp region contained 28 CpG sites (Figure 1), of which the methylation status of 24 sites could be measured by EpiTYPER® technology. For certain adjacent CpG sites, EpiTYPER® could only report their methylation status as one unit. We therefore arbitrarily refer to all successfully measured CpG loci as units. A total of 17 CpG units passed our stringent quality control criteria (see Materials and Methods for details). CpG units within the first exon exhibited generally higher methylation levels than those upstream of the TSS.

### Genetic analysis of CpG methylation with MetS traits in our family cohort

Table 2 shows the heritability of the methylation status at each of the 17 CpG units that we successfully profiled. We found that except for three CpG units (2, 3 and 17/18), the methylation patterns of these units are genetically shared by family members, although their heritability levels varied greatly from 0.15 to 0.48. We tested for the effect of age and sex on methylation status of these units and found that the methylation level at several units is affected by age and sex. For example, the methylation status of site 9 is positively correlated with age (βage = 2.90E-04, p = 3.95E-05) whereas CpG site 19 was influenced by sex (βsex = 1.30E-02, p = 3.88E-04). For these CpG loci, the influence of age on their methylation levels is different from that of sex, with some units showing a greater influence of age and others being affected more by sex.

**Table 2:** **Heritability and correlations with age and sex of methylation status near *FABP3 *start codon**

**CpG site**	**Heritability ±SE**	**P**_**heritability**_	**β±SE**_**age**_	**P**_**age**_	**Variance explained **_**age**_	**β±SE**_**sex**_	**P**_**sex**_	**Variance explained **_**sex**_
FABP3_1	0.21 ± 0.07	2.85E-05	1.70E-04 ± 1.00E-04	1.03E-01	2.99E-03	1.90E-03 ± 3.50E-03	5.87E-01	2.19E-04
FABP3_2	0.05 ± 0.07	1.89E-01	1.60E-04 ± 4.90E-05	1.64E-03	1.03E-02	−7.30E-04 ± 1.60E-03	6.59E-01	1.98E-04
FABP3_3	0.09 ± 0.08	1.11E-01	1.50E-04 ± 6.20E-05	1.32E-02	1.41E-02	4.60E-03 ± 2.00E-03	2.34E-02	5.55E-03
FABP3_4	0.18 ± 0.08	4.39E-03	4.00E-05 ± 5.00E-05	4.11E-01	1.27E-03	2.70E-03 ± 1.60E-03	8.93E-02	3.10E-03
FABP3_5	0.15 ± 0.07	7.38E-03	1.10E-04 ± 3.20E-05	7.44E-04	1.41E-02	5.40E-04 ± 1.00E-03	6.06E-01	7.51E-05
FABP3_6/7	0.17 ± 0.08	5.15E-03	1.20E-04 ± 3.20E-05	1.09E-04	1.52E-02	2.50E-04 ± 1.10E-03	8.11E-01	2.61E-05
FABP3_8	0.17 ± 0.08	3.23E-03	−2.00E-05 ± 9.80E-05	8.35E-01	3.09E-04	6.20E-03 ± 3.20E-03	5.23E-02	3.44E-03
FABP3_9	0.48± 0.08	4.53E-17	2.90E-04 ± 7.10E-05	3.95E-05	2.09E-02	−7.60E-04 ± 2.40E-03	7.48E-01	1.31E-04
FABP3_10/11/12	0.19 ± 0.09	4.40E-03	2.80E-04 ± 6.80E-05	5.77E-05	2.05E-02	6.00E-03 ± 2.20E-03	6.55E-03	7.31E-03
FABP3_13	0.46 ± 0.10	1.06E-09	2.20E-04 ± 1.10E-04	3.75E-02	8.40E-03	3.90E-03 ± 3.50E-03	2.66E-01	1.58E-03
FABP3_14	0.48 ± 0.10	2.41E-09	2.80E-04 ± 1.30E-04	3.51E-02	7.52E-03	1.30E-02 ± 4.40E-03	2.50E-03	1.11E-02
FABP3_15	0.15 ± 0.07	3.92E-03	−2.80E-04 ± 1.40E-04	4.77E-02	4.15E-03	4.50E-03 ± 4.60E-03	3.34E-01	9.95E-04
FABP3_16	0.32 ± 0.10	1.90E-05	2.30E-04 ± 8.70E-05	8.20E-03	1.04E-02	6.30E-03 ± 2.80E-03	2.72E-02	5.63E-03
FABP3_17/18	0.03 ± 0.06	2.80E-01	1.30E-04 ± 1.40E-04	3.49E-01	9.21E-04	7.60E-03 ± 4.70E-03	1.03E-01	2.78E-03
FABP3_19	0.26 ± 0.09	5.76E-05	−3.30E-04 ± 1.10E-04	2.97E-03	5.46E-03	1.30E-02 ± 3.60E-03	3.88E-04	1.16E-02
FABP3_20	0.41 ± 0.09	4.52E-09	1.00E-05 ± 9.30E-05	8.79E-01	2.61E-05	8.10E-03 ± 3.10E-03	8.91E-03	3.88E-03
FABP3_21/22	0.14± 0.08	2.15E-02	−7.00E-05 ± 1.60E-04	6.34E-01	5.56E-04	−5.50E-03 ± 5.40E-03	3.06E-01	2.50E-03
FABP3_AVG	0.22 ± 0.07	9.35E-06	9.30E-04 ± 6.10E-04	1.24E-01	5.00E-03	6.00E-02 ± 2.00E-02	2.23E-03	5.99E-03

Heritability (±SE) estimates are shown for the 17 individual CpG units and their average. Significance p-values for heritability estimates are shown. Association β (±SE) values, their associated significance p-values, and the portion of total variance in methylation explained by age and sex are also shown.

**Figure 2:**
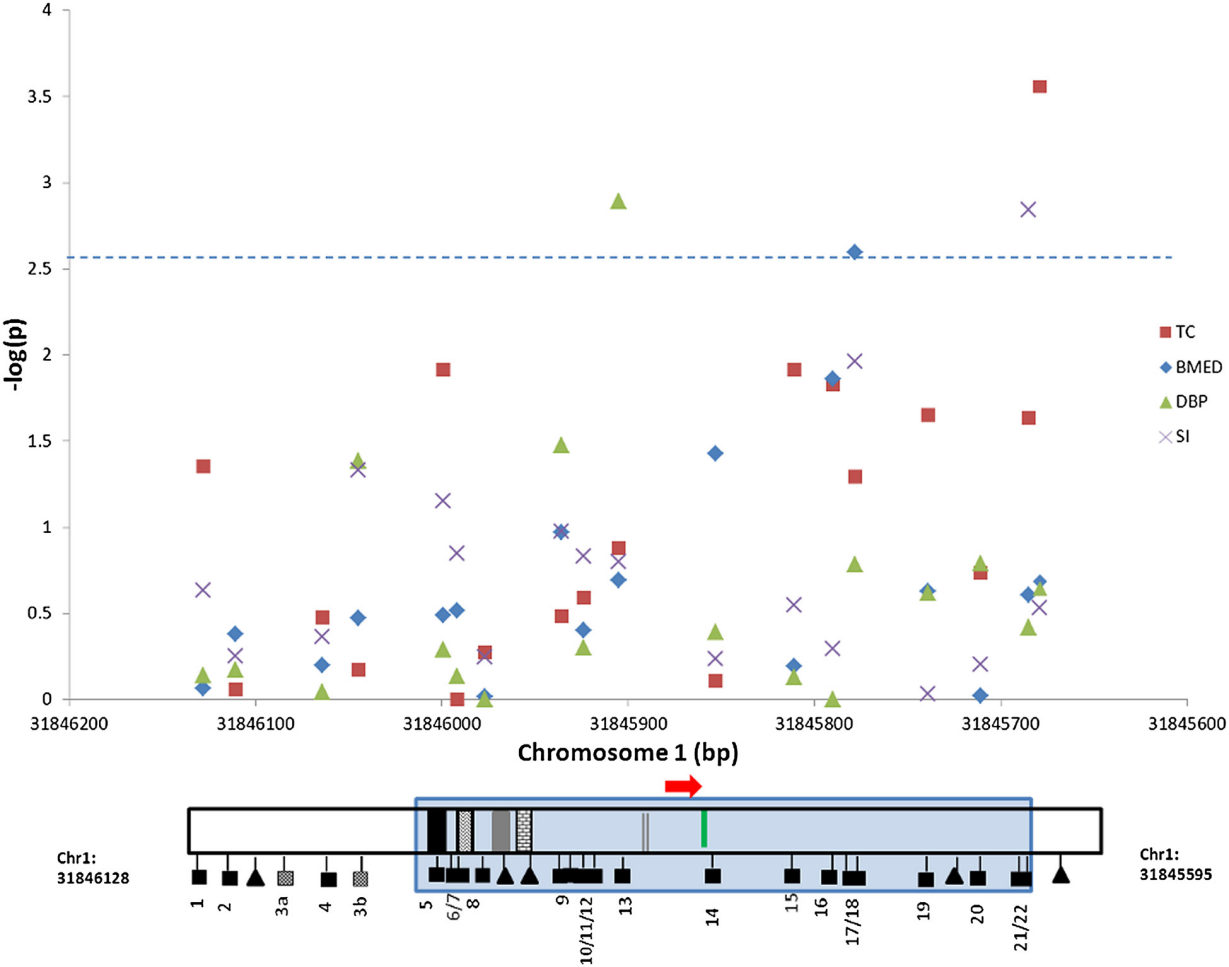
**Manhattan plot of CpG methylation status in association with MetS traits.** P-values for traits that were at least suggestively significantly associated with methylation at one or more CpG sites [presented as –log(p) on the y-axis], were plotted against the chromosomal positions of analyzed CpG sites. *FABP3* is encoded on the - strand so bp locations (annotated to the + strand) are displayed in reverse order. Plot symbol shapes represent specific MetS traits (see right margin of each panel). Symbols represent association of the 17 individual CpG loci as well as the regional average (arbitrarily assigned to a position after the last CpG site). The gene architecture of the region is shown under the plots. The dashed line represents the statistical significance threshold (blue, alpha=0.05) after correction for multiple testing.

We then tested for association of quantitative methylation levels of these 17 CpG units, as well as average methylation across the entire region assessed, with 42 MetS phenotypes. Phenotypes that were significantly associated (p<0.0028, alpha = 0.05 corrected for 18 CpG loci; p_α=0.05_=6.7×10^-5^, if all 42 MetS phenotypes are treated as non-independent traits in one hypothesis) with the methylation status of at least one site or the regional average are shown in Table 3 and Figure 2. The strongest association was that between overall average methylation and total cholesterol level (p=0.00028). Methylation at units 13 and 21/22 was associated with diastolic blood pressure (p=0.00129) and insulin sensitivity (p=0.0014), respectively. Methylation of site 17/18 within the first exon was associated with apoB-containing non-HDL particle size (BMED), which includes VLDL, ILDL, LPα and LDL (p = 0.0025). Regional average methylation was also suggestively associated (p<0.0056, alpha = 0.10 corrected for 18 tests) with LDL cholesterol (p = 0.0050). We observed that the proportion of trait variance that can be explained by associated CpG methylation status is comparable to previously reported contributions of individual SNPs to the disease trait (1-3%) (Table 3). We also estimated the back transformed absolute value in each associated MetS phenotype per percentile change in methylation of particular CpG loci. For instance, we found for every 1% increase in regional average methylation of *FABP3*, the plasma level of total cholesterol was estimated to increase by 6.60 mg/dl.

**Table 3:** Associations between MetS traits and methylation status of FABP3

**MetS trait**	**CpG Site**	**β±SE**	**Var explained**	**Change attributable to methylation**	**P value**
TC (mg/dl)	FABP3_AVG	0.17* ± 0.045	0.0240	6.60 ± 0.045	0.00028
BMED (nm)	FABP3_17/18	−0.15* ± 0.05	0.0210	−0.18 ± 0.051	0.00253
DBP (mmHg)	FABP3_13	−0.16* ± 0.049	0.0170	−1.65 ± 0.050	0.00129
SI (× 10-4/min/μU/ml)	FABP3_21/22	−0.16* ± 0.051	0.0160	−0.39 ± 0.051	0.00144

The traits or CpG units that showed any significant associations are presented. For each trait/CpG pair, β (±SE), total trait variance explained by the association (varexp), and the absolute change in that trait attributable to methylation of the specific CpG site are shown. *associations with confidence interval of 95%.

### Genetic analysis of the relationship between PWBC expression of FABP3 and MetS traits related to lipids and adipokines in the expression study cohort

In parallel to the methylation analysis of FABP3 in relation to the MetS traits in our family cohort, we analyzed the expression levels of this gene in PWBCs in a separate group of subjects from our MRC-OB collections. Table 4 shows the sex-specific means (±standard deviation, STD) of the 15 MetS phenotypes measured in 128 individuals of 55 extended families of Northern European descent. 52.3% of the cohort is female, the average age of the cohort is 41.1y and the average BMI is 31.95 kg/m^2^.

**Table 4:** Phenotypic characteristics of the expression cohort

**Trait**	**Mean ± STD (Male)**	**Mean ± STD (Female)**
Weight, kg	98.26 ± 24.01	88.43 ± 25.48
Height, cm	176.77 ± 7.35	165.17 ± 7.21
BMI, kg/m_2_	31.41 ± 7.37	32.44 ± 9.24
Waist circumference (WC), cm	96.25 ± 16.62	91.39 ± 20.47
Hip circumference (HC), cm	96.53 ± 17.13	100.91 ± 20.99
Waist to Hip ratio (WHR)	1.00 ± 0.11	0.91 ± 0.12
Fasting Glucose (FG), mg/dl	86.45 ± 23.69	88.41 ± 27.29
Fasting Insulin (FI),μU/ml	21.79 ± 22.60	19.02 ± 10.65
Homeostasis model assessment (HOMA)	4.72 ± 4.92	4.51 ± 4.04
Triglycerides (TG), mg/dl	117.53 ± 63.98	95.09 ± 41.08
Total cholesterol (TC), mg/dl	183.82 ± 36.36	182.10 ± 36.03
LDL-cholesterol (LDL-c), mg/dl	126.62 ± 33.69	121.93 ± 32.51
HDL-cholesterol (HDL-c), mg/dl	40.58 ± 10.50	47.64 ± 14.58
Adiponectin, ng/ml	10.04 ± 7.63	11.82 ± 5.59
Leptin, ng/ml	9.25 ± 9.04	24.87 ± 21.39

The mean +/− standard deviation is shown for each of the MetS phenotypes.

Specifically, we analyzed in our family cohort the relationship of levels of FABP3 transcripts in PWBCs with quantitative measures of MetS phenotypes focusing on the lipids, fat distribution (WHR) and plasma levels of two key protein hormone exclusively produced by adipose tissue (adiponectin and leptin). Table 5 shows the phenotype-specific correlation beta values (±standard error) of FABP3 expression in PWBCs. In general, the gene expression of FABP3 correlated positively with MetS phenotypes that are known to have beneficial effects in metabolic profiles including HDL-cholesterol (β_HDL-c_ = 0.42) and adiponectin (β_adiponectin_ = 0.24). In contrast, FABP3 expression in the PWBCs correlated negatively with TC (β_TC_ = −0.25), LDL-c (β_LDL-c_ = −0.53), TG (β_TG_ = −0.20), and WHR (β_WHR_ = −0.72), whose elevation is usually linked with an unhealthy profile of metabolism.

**Table 5:** **Correlations of *FABP3 *gene expression with MetS traits related to lipids and adipokines**

**MetS trait**	**β±SE**	**P value**
TC, mg/dl	−0.25 ± 0.31	0.43
LDL-c, mg/dl	−0.53 ± 0.31	0.09
HDL-c, mg/dl	0.42 ± 0.32	0.18
TG, mg/dl	−0.20 ± 0.33	0.54
WHR	−0.72 ± 0.37	0.05
Adiponectin, ng/ml	0.24 ± 0.39	0.53
Leptin, ng/ml	−0.08 ± 0.46	0.86

For each trait, the β (±SE) and the p-value are shown.

### The methylation status of the concise promoter affects FABP3 binding of heart nuclear proteins

**Figure 3:**
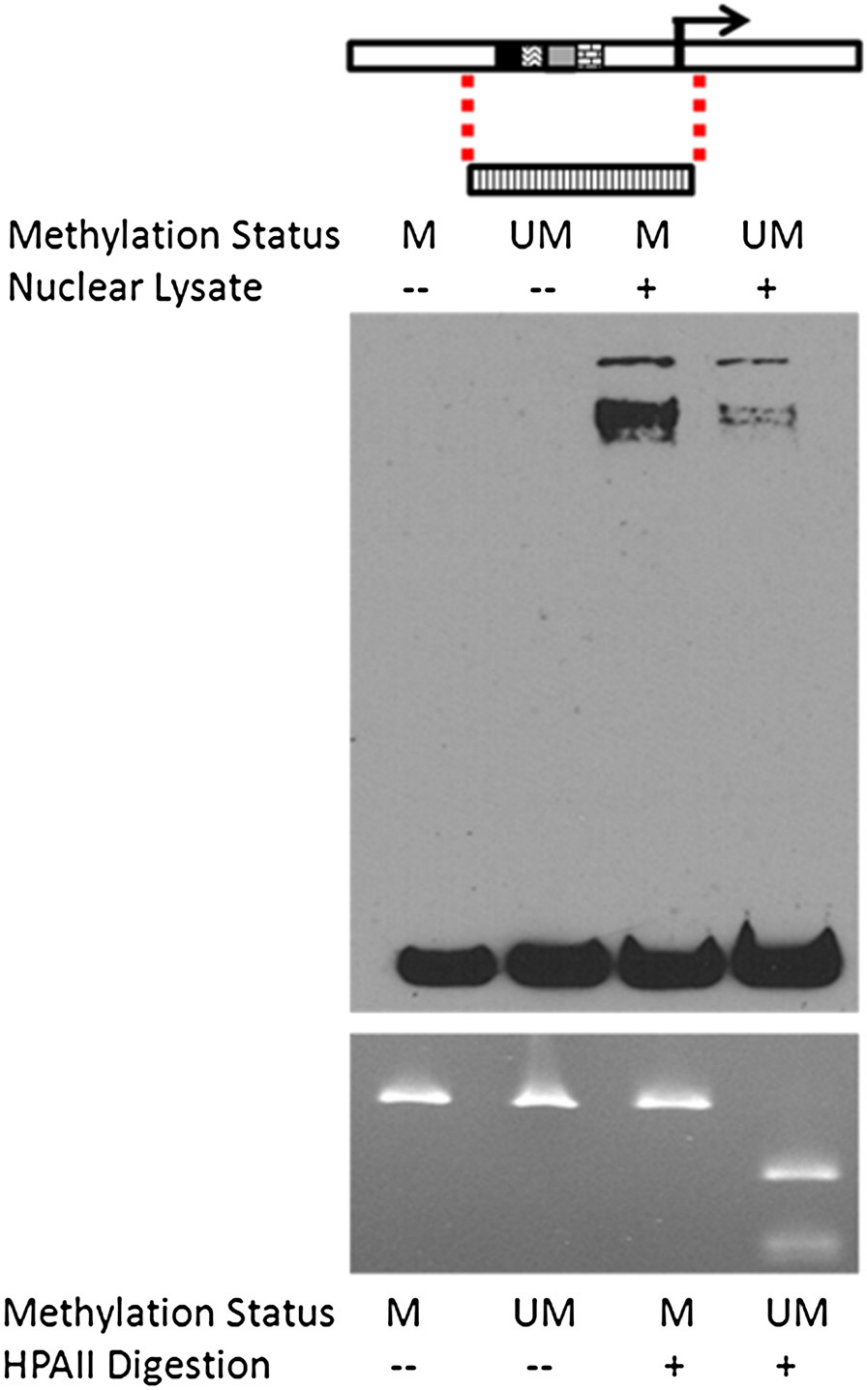
**EMSA of differentially methylated *FABP3 *promoter region.** Completely methylated (M) or completely unmethylated (UM), biotin-tagged DNA fragments were assayed for binding activity using nuclear extract from human left ventricle tissue. The location of the amplified region is depicted by the graph on the top panel. The middle panel shows the EMSA results with biotin-labeled free DNA at the bottom and protein-bound DNA at the top. The lower panel shows UV fluorescence of the E-Gel® fluorescent stain (Invitrogen) (M or UM) after methylation-sensitive restriction enzyme digestion by HPAII. Results are representative of at least three independent experiments.

We hypothesized that CpG methylation status is able to influence the expression of FABP3 by modifying the binding affinity of the region for specific transcription factors. We tested this hypothesis by performing a methylation-specific EMSA using nuclear extract from human left ventricle tissue. In this EMSA, instead of interrogating the binding capacity of the two alternative nucleotides of a SNP as is routinely done in an EMSA experiment, we applied it to completely methylated and completely unmethylated promoter regions, that were chosen based upon the location of previously determined transcription elements (53). As shown in Figure 3, in the presence of heart nuclear proteins, two bands shifted from the pool of unbound biotin-labeled DNA probes to higher molecular weight, suggesting nuclear protein(s) from this tissue can bind with the FABP3 promoter sequence. Further, completely methylated DNA sequences bound with higher affinity to these unknown protein factors than unmethylated DNA sequences, suggesting that differential methylation of the FABP3 promoter affects its binding with nuclear factors that could regulate its gene expression in heart. Our observed pattern suggests elevated levels of FABP3 promoter methylation could recruit negative regulators of transcription, as higher levels of CpG methylation of a gene promoter has been associated with gene repression [54,55].

## Discussion

We conducted a statistical analysis of the hypothesized association of epigenetic modifications of FABP3 gene promoter with MetS traits, in a family-based population of Northern European ancestry. We found that methylation states in blood cell-derived DNA are significantly heritable in our families, and the methylation of several individual CpG units as well as the regional average methylation is significantly associated with MetS traits. We also found that differential methylation at these units affects the affinity of this region of DNA for binding to nuclear proteins extracted from human heart, thus indicating a possible mechanism by which methylation may regulate gene-expression in this region. The CpG island we identified near the transcription start site of *FABP3* is a 533 bp region spanning the promoter region and part of the first exon. There is evidence that expression levels of various genes are influenced not just by methylation of the promoter region, but also by methylation status of the early exons and introns [33,34]. In our study, we found that methylation levels within the *FABP3* promoter region were low (from 0.6% to 7.9%), but were dramatically higher (up to 95.6%) in the first exon. Heritability appears to be strong for a majority of the individual CpG units in our families; both in the promoter region and in the first exon, suggesting that propensity for methylation at certain genomic loci can be transmitted from generation to generation.

The proportion of variance in site specific methylation that can be explained by age or sex was found to be moderate with the highest effect size being approximately 2%, suggesting methylation may be influenced more so by genetic (as our heritability estimates suggest) and environmental factors. Still, while their effect sizes were modest, age and sex did have a statistically significant effect on methylation status at a number of CpG units. *FABP3*_ 10/11/12, 14, 16 and 19 appeared to be affected by both age and sex, whereas several units such as *FABP3*_2, 5, 6, 9 and 15, were only correlated with age but not sex, and others, like *FABP3*_4, 8 and 20 were influenced by sex but not by age. These results demonstrate the complexity of epigenetic modifications and their interaction with age and sex. Because the limitation inherent in the EpiTYPER technology, some closely located CpG sites were detected and reported as one methylation “unit”. This needs to be considered when interpreting the information of sites that were not distinguished as individual elements.

There is evidence that FABPs play the following roles in tissue-targeted fatty acid management: assisting uptake and trafficking of long-chain fatty acids (LFCAs); targeting LCFAs for metabolic pathways (in muscle); mediating lipid-mediated gene expression (adipose tissue and macrophages); maintaining an intracellular pool of LCFAs as cell fuel (mainly in muscle); and protecting cellular proteins and membranes from the toxicity of LCFAs and their derivatives [3]. Alterations in the function of FABPs can therefore impact lipid metabolism and cellular function in diverse tissues. Alterations in *FABP3* in particular, are associated with altered lipid metabolism and cardiac and hepatic abnormalities in animal models. Mice lacking H-FABP showed a severe defect in utilizing peripheral (non-fat and nonhepatic) LCFAs [10]. In these mice the heart was forced to switch to glucose fuel and the liver was burdened with increased loads of LCFA oxidation. This H-FABP deficiency made the heart intolerant to acute exercise and eventually led to localized cardiac hypertrophy. In H-FABP mutant mice models, exercise-induced elevation in metabolic demands is dependent upon H-FABP to reciprocally balance glucose and LCFA utilization in multiple tissues of the body [11]. Thus there are several potential mechanisms by which alteration in *FABP3* function could affect lipid metabolism and cardiovascular function in humans.

We found that the methylation level at several individual units as well as the average methylation level in the region is associated with variation in multiple metabolic syndrome phenotypes. Phenotypes that were associated with *FABP3* methylation included plasma lipids (TC, LDL-c), lipoprotein sizing (BMED), blood pressure (dBP), insulin sensitivity (SI). The size of these effects, while generally less than 3%, is large enough to be of clinical significance (for example, we found a mean variation of 6.60 mg/dl in total cholesterol for each 1% change in average total methylation) and is comparable to the effect sizes of single nucleotide polymorphisms (SNPs) on complex disease traits [56,57]. In our study, we have found that the variation in a Mets phenotype that can be contributable to individual CpG site methylation is comparable to the estimated effect sizes of an individual SNP to a similar trait from previous studies. This suggests that in this complex disease model, genomic DNA methylation variation contribute in a similar way as SNP variants do. That might be the reason that we observe individual site contributes to only a small portion of the total phenotype variance. Clinically however, this is meaningful because the changes in mean levels of various risk factors are usually small, but even small changes in mean levels can have a measurable benefit at a population wide level [58]. Most intervention trials using diet and drugs report mean alterations comparable to those seen in our population, but these are associated with significant decline in the incidence of cardiovascular and other diseases [59,60]. In the case of lipid phenotypes, the mechanism of these effects may be directly related to the role of FABPs in lipid metabolism. In addition, since FABPs may play a central role in coordinating inflammatory and metabolic responses in the cell, an alteration in gene function could influence the levels of inflammatory cytokines and induce insulin resistance. An effect on blood pressure has been reported in an earlier study and may be mediated via insulin resistance in skeletal muscle [21]. Taken together, these observations suggest that alterations in methylation of the *FABP3* promoter region can be plausibly linked to various biological precursors of metabolic syndrome. Further studies will be conducted to ascertain how methylation status is related to gene expression and downstream physiologic effects in target tissues and animal models.

Since promoter CpG methylation is known to affect the function of a gene by influencing the expression, we conducted an analysis of correlations between the transcript levels of FABP3 and several key MetS phenotypes pertinent to lipid metabolism. Our data showed that increased levels of FABP3 are correlated with measures indexing a beneficial metabolic profile whereas decreased levels of FABP3 are correlated with metabolic profiles increase individual’s risk for developing cardiovascular diseases (CVDs) [61,62]. This suggests that increased levels of FABP3 may benefit one’s metabolic profiles. If taken together with the results of FABP3 promoter methylation association with related traits, our data show a consistent pattern in which lipid traits such as plasma total cholesterol, correspond to higher methylation levels of FABP3 at the promoter and lower expression levels of FABP3 in our surrogate tissue PWBCs. A future study of bigger sample sizes may help strengthen the statistical significance of the signals we obtained.

We further explored whether differential methylation states of the core promoter region of FABP3, which a previous study had shown to be essential for the regulation of the expression of this gene (53), could affect the binding of transcription factors in a tissue of interest, the heart. Our gel shift assay showed a clear difference between completely methylated and completely unmethylated fragments of the gene promoter region. This suggests a molecular mechanism for the associations that differential methylation of FABP3 promoter alters binding capacity of nuclear proteins that regulates FABP3 expression, which in turn leads to variation in MetS phenotypes. The pattern we observed here indicates that increased methylation state of this region could contribute to the recruitment of transcription repressors, if FABP3 transcription is under the canonical regulation model where denser methylation correspond to repressed transcription levels (54, 55). Our assay was designed as a proof of concept to test the extreme scenario of completely methylated region of interest in comparison to its completely unmethylated counterpart, however it may not reflect the endogenous physiologic conditions. This assay also does not demonstrate the precise site that these factors bind to, nor did it identify those factors. A study that addresses these questions could provide valuable information and will be pursued in the future.

Evidence recently published by the ENCODE project demonstrated the extensiveness and universality of elements of the functional genome [63] and epigenetic tags including CpG methylation and histone modification codes are of pivotal importance in functional genomics regulation [64-66]. We have found that in K562, a leukemia cell line representing features of lymphocytes, the promoter region of *FABP3* that we investigated overlaps with a region of peak signals for H3K4me1 and H3K9ac, the two histone modification markers that are usually indicative of active regulatory chromatin domain for target gene expression [67-72]. This data together with ours, suggest *FABP3* is under complex epigenetic regulation in the cell. Further studies that address these novel aspects in relation to phenotype expression may be of importance to advance our understanding of pathogenesis of MetS traits.

## Conclusions

In summary, the current study provides evidence that the methylation status of *FABP3* is associated with phenotypes of metabolic syndrome. Our evidence characterizing the heritability, age and sex effects on high resolution CpG methylation provides initial insights into the epigenetic regulation of an important biological gene in humans. More research is needed to replicate our findings and to elucidate the mechanisms and physiologic pathways through which methylation status affects these phenotypes representative of cardiovascular function, lipid metabolism and insulin sensitivity. Our work demonstrating the clinical relevance of the epigenetic markers of an important biological candidate such as *FABP3* can be a step towards clinical tests that measure epigenetic alterations that can predict patients’ risks of developing insulin, lipids and blood pressure phenotypes of MetS.

## Abbreviations

FABP3: Fatty acid-binding protein gene; EMSA: Electrophoretic mobility shift assay; LCFAs: Long-chain fatty acids; SAFHS: San Antonio Family Heart Study; MetS: Metabolic syndrome; CVD: Cardiovascular disease; MBD: Methyl-binding domain; SNP: Single nucleotide polymorphism; BMI: Body Mass Index; WC: Waist circumference; HC: Hip circumference; WHR: Waist to hip ratio; FG: Fasting glucose; FI: Fasting insulin; IGR: Insulin to glucose ratio; HOMA: Homeostasis model assessment; TG: Plasma triglycerides; TC: Total cholesterol; LDL-c: LDL-cholesterol; cal. LDL-c: Calculated LDL-cholesterol levels; HDL-c: HDL-cholesterol; sBP: Systolic blood pressure; dBP: Diastolic blood pressure; Fatkg: Total fat mass in kilogram; Fatpct: Total fat mass in percentage; Leankg: Lean mass in kilogram; Leanpct: Lean mass in percentage; TAF: Total abdominal fat size; VF: Visceral fat size; SubQF: Subcutaneous fat size; REE: Resting energy expenditure; RQ: Respiratory quotient; SI: Insulin sensitivity; SG: Glucose effectiveness; AIRG: Acute insulin response to glucose; DI: Disposition index; HMED: HDL median diameter; LMEDn: LDL-cholesterol median diameter; LDLppd: LDL-cholesterol dominant peak diameter; BMED: ApoB-containing non-HDL median diameter; (BSM): Bisulfite modified; SAP: Shrimp alkaline phosphatase.

## Competing interests

The authors declare that they have no competing interests.

## Authors’ contributions

Dr. AK was fully involved in the preparation of this report up to his death on 17 May 2012. We are saddened by his loss and deeply respectful of his long and distinguished career in obesity research. YZ conceived of the study, participated in the design of the study, led and participated in all molecular procedures and the statistical analysis, drafted and edited the manuscript. JWK performed all procedures of the statistical analysis, participated in the discussion and edited the manuscript. AL participated in epigenetic profiling and facilitated the discussion. DC participated in carrying out molecular assays. OA facilitated the discussion and edited the manuscript. RD facilitated in the execution of the study and the discussion. MO participated in the design of the study, facilitated in the execution of the study, participated in the discussion and reviewed the manuscript. JB participated in the statistical analysis, participated in the design and discussion of the study, and reviewed the manuscript. MAC participated in the design, execution of the study, participated in the discussion and revised the manuscript. AHK participated in the design, execution, and the discussion of the study. All authors read and approved the final manuscript.

## Pre-publication history

The pre-publication history for this paper can be accessed here:

http://www.biomedcentral.com/1755-8794/6/9/prepub

## Supplementary Material

Additional file 1: Table S1 PCR Primers for Methylation Detection of the Promoter and First Exon region of FABP3. *Forward Primers contain the T7 promoter tag (5^′^ AGG AAG AGA G 3^′^) on the 5^′^ end of the primer sequence. **Reverse primers contain the T7-promoter tag (5^′^ CAG TAA TAC GAC TCA CTA TAG GGA GAA GGC T 3^′^) on the 5^′^ end of the primer sequence. Primers are complementary to the reverse (-) template strand. Click here for file

## References

[B1] Khan SA Vanden Heuvel JP Reviews: current topics role of nuclear receptors in the regulation of gene expression by dietary fatty acids (review) J Nutr Biochem 2003 14 554 567 10.1016/S0955-2863(03)00098-6 14559106

[B2] Day C Metabolic syndrome, or What you will: definitions and epidemiology Diab Vasc Dis Res 2007 4 32 38 10.3132/dvdr.2007.003 17469041

[B3] Storch J Thumser AE Tissue-specific Functions in the Fatty Acid-binding Protein Family J Biol Chem 2010 285 32679 32683 10.1074/jbc.R110.135210 20716527 PMC2963392

[B4] Makowski L Hotamisligil GS The role of fatty acid binding proteins in metabolic syndrome and atherosclerosis Curr Opin Lipidol 2005 16 543 548 10.1097/01.mol.0000180166.08196.07 16148539 PMC3904771

[B5] Xu A Wang Y Xu JY Stejskal D Tam S Zhang J Wat NM Wong WK Lam KS Adipocyte fatty acid-binding protein is a plasma biomarker closely associated with obesity and metabolic syndrome Clin Chem 2006 52 405 413 10.1373/clinchem.2005.062463 16423904

[B6] Akbal E Özbek M Güneş F Akyürek O Üreten K Delibaş T Serum heart type fatty acid binding protein levels in metabolic syndrome Endocrine 2009 36 433 437 10.1007/s12020-009-9243-6 19806479

[B7] Heuckeroth RO Birkenmeir EH Levin MS Gordon JI Analysis of the Tissue-specific Expression, Developmental Regulation, and Linkage Relationships of a Rodent Gene Encoding Heart Fatty Acid Binding Protein J Biol Chem 1987 262 9709 9717 3036869

[B8] Veerkamp JH Paulussen RJA Peeters RA Maatman RGHJ Van Moerkert HTB Van Kuppevelt TH Detection, tissue distribution, and (sub)cellular localization of fatty acid binding protein types Mol Cell Biochem 1990 98 11 18 2266952 10.1007/BF00231362

[B9] Su AI Wiltshire T Batalov S Lapp H Ching KA Block D Zhang J Soden R Hayakawa M Kreiman G Cooke MP Walker JR Hogenesch JB A gene atlas of the mouse and human protein-encoding transcriptomes Proc Natl Acad Sci USA 2004 101 6062 6067 10.1073/pnas.0400782101 15075390 PMC395923

[B10] Binas B Dannenberg H McWhir J Mullins L Clark JA Requirement for the heart-type fatty acid binding protein in cardiac fatty acid utilization FASEB J 1999 13 805 812 10224224 10.1096/fasebj.13.8.805

[B11] Shearer J Fueger PT Rottman JN Bracy DP Binas B Wasserman DH Heart-type fatty acid-binding protein reciprocally regulates glucose and fatty acid utilization during exercise Am J Physiol Endocrinol Metab 2005 288 E292 E297 10.1152/ajpendo.00287.2004 15454399

[B12] Li B Zerby HN Lee K Heart fatty acid binding protein is upregulated during porcine adipocyte development J Anim Sci 2007 85 1651 1659 10.2527/jas.2006-755 17431053

[B13] Shearer J Fueger PT Bracy DP Wasserman DH Rottman JN Partial gene deletion of heart-type fatty acid-binding protein limits the severity of dietary-induced insulin resistance Diabetes 2005 54 3133 3139 10.2337/diabetes.54.11.3133 16249436

[B14] Shioda N Yamamoto Y Watanabe M Binas B Owada Y Fukunaga K Heart-Type Fatty Acid Binding Protein Regulates Dopamine D_2_ Receptor Function in Mouse Brain J Neurosci 2010 30 3146 3155 10.1523/JNEUROSCI.4140-09.2010 20181611 PMC6633935

[B15] Niizeki T Takeishi Y Takabatake N Shibata Y Konta T Kato T Kawata S Kubota I Circulating levels of heart-type fatty acid-binding protein in a general Japanese population: effects of age, gender, and physiologic characteristics Circ J 2007 71 1452 1457 10.1253/circj.71.1452 17721027

[B16] Karbek B Özbek M Bozkurt NC Ginis Z Güngünes A Ünsal IÖ Cakal E Delibası T Heart-type fatty acid binding protein (H-FABP): relationship with arterial intima-media thickness and role as diagnostic marker for atherosclerosis in patients with ımpaired glucose metabolism Cardiovasc Diabetol 2011 10 37 10.1186/1475-2840-10-37 21535886 PMC3112391

[B17] Watanabe K Wakabayashi H Veerkamp JH Ono T Suzuki T Immunohistochemical distribution of heart-type fatty acid binding protein immunoreactivity in normal human tissues and acute myocardial infarction J Pathol 1993 170 59 65 10.1002/path.1711700110 8326460

[B18] Grundy SM Brewer HB Jr Cleeman JI Smith SC Jr Lenfant C American Heart Association; National Heart, Lung, and Blood Institute. Definition of metabolic syndrome: Report of the National Heart, Lung, and Blood Institute/American Heart Association conference on scientific issues related to definition Circulation 2004 109 433 438 10.1161/01.CIR.0000111245.75752.C6 14744958

[B19] Evans DJ Hoffmann RG Kalkhoff RK Kissebah AH Relationship of body fat topography to insulin sensitivity and metabolic profiles in premenopausal women Metabolism 1984 33 68 75 10.1016/0026-0495(84)90164-1 6361449

[B20] Peiris AN Mueller RA Smith GA Struve MF Kissebah AH Splanchnic insulin metabolism in obesity. Influence of body fat distribution J Clin Invest 1986 78 1648 1657 10.1172/JCI112758 3537010 PMC423938

[B21] Ueno T Soma M Tabara Y Tokunaga K Tahira K Fukuda N Matsumoto K Nakayama T Katsuya T Ogihara T Makita Y Hata A Yamada M Takahashi M Hirawa N Umemura S Miki T Association between fatty acid binding protein 3 gene variants and essential hypertension in humans Am J Hypertens 2008 21 691 695 10.1038/ajh.2008.40 18437121

[B22] Shin HD Kim LH Park BL Jung HS Cho YM Moon MK Lee HK Park KS Polymorphisms in fatty acid-binding protein-3 (FABP3) – putative association with type 2 diabetes mellitus Hum Mutat 2003 22 180 12872269 10.1002/humu.9168

[B23] Murphy SK Jirtle RL Imprinting evolution and the price of silence BioEssays 2003 25 577 588 10.1002/bies.10277 12766947

[B24] Portela A Esteller M Epigenetic modifications and human disease Nat Biotechnol 2010 28 1057 1068 10.1038/nbt.1685 20944598

[B25] Sharma S Kelly TK Jones PA Epigenetics in cancer Carcinogenesis 2010 31 27 36 10.1093/carcin/bgp220 19752007 PMC2802667

[B26] Lillycrop KA Phillips ES Jackson AA Hanson MA Burdge GC Dietary protein restriction of pregnant rats induces and folic acid supplementation prevents epigenetic modification of hepatic gene expression in the offspring J Nutr 2005 135 1382 1386 15930441 10.1093/jn/135.6.1382

[B27] Bogdarina I Welham S King PJ Burns SP Clark AJ Epigenetic modification of the renin-angiotensin system in the fetal programming of hypertension Circ Res 2007 100 520 526 10.1161/01.RES.0000258855.60637.58 17255528 PMC1976252

[B28] Lillycrop KA Slater-Jefferies JL Hanson MA Godfrey KM Jackson AA Burdge GC Induction of altered epigenetic regulation of the hepatic glucocorticoid receptor in the offspring of rats fed a protein-restricted diet during pregnancy suggests that reduced DNA methyltransferase-1 expression is involved in impaired DNA methylation and changes in histone modifications Br J Nutr 2007 97 1064 1073 10.1017/S000711450769196X 17433129 PMC2211425

[B29] Fan S Zhang X CpG island methylation pattern in different human tissues and its correlation with gene expression Biochem Biophys Res Commun 2009 383 421 425 10.1016/j.bbrc.2009.04.023 19364493

[B30] Scarpellini E Tack J Obesity and metabolic syndrome: an inflammatory condition Dig Dis 2012 30 2 148 153 10.1159/000336664 22722429

[B31] Hotamisligil GS **Endoplasmic reticulum stress and the inflammatory basis of metabolic** disease Cell 2010 140 900 917 10.1016/j.cell.2010.02.034 20303879 PMC2887297

[B32] Neels JG Olefsky JM Inflamed fat: what starts the fire? J Clin Invest 2006 116 33 35 16395402 10.1172/JCI27280 PMC1323268

[B33] Toperoff G Aran D Kark JD Rosenberg M Dubnikov T Nissan B Wainstein J Friedlander Y Levy-Lahad E Glaser B Hellman A Genome-wide survey reveals predisposing diabetes type 2-related DNA methylation variations in human peripheral blood Hum Mol Genet 2012 21 371 383 10.1093/hmg/ddr472 21994764 PMC3276288

[B34] Bell CG Finer S Lindgren CM Wilson GA Rakyan VK Teschendorff AE Akan P Stupka E Down TA Prokopenko I Morison IM Mill J Pidsley R Deloukas P Frayling TM Hattersley AT McCarthy MI Beck S Hitman GA International Type 2 Diabetes 1q Consortium Integrated genetic and epigenetic analysis identifies haplotype-specific methylation in the FTO type 2 diabetes and obesity susceptibility locus PLoS One 2010 5 e14040 10.1371/journal.pone.0014040 21124985 PMC2987816

[B35] Jiang MH Fei J Lan MS Lu ZP Liu M Fan WW Gao X Lu DR Hypermethylation of hepatic Gck promoter in ageing rats contributes to diabetogenic potential Diabetologia 2008 51 1525 1533 10.1007/s00125-008-1034-8 18496667

[B36] Ling C Del Guerra S Lupi R Ronn T Granhall C Luthman H Masiello P Marchetti P Groop L Del Prato S Epigenetic regulation of PPARGC1A in human type 2 diabetic islets and effect on insulin secretion Diabetologia 2008 51 615 622 10.1007/s00125-007-0916-5 18270681 PMC2270364

[B37] Mitchell BD Kammerer CM Blangero J Mahaney MC Rainwater DL Dyke B Hixson JE Henkel RD Sharp RM Comuzzie AG VandeBerg JL Stern MP MacCluer JW Genetic and Environmental Contributions to Cardiovascular Risk Factors in Mexican Americans: The San Antonio Family Heart Study Circulation 1996 94 2159 2170 10.1161/01.CIR.94.9.2159 8901667

[B38] Smith EM Zhang Y Baye TM Gawrieh S Cole R Blangero J Carless MA Curran JE Dyer TD Abraham LJ Moses EK Kissebah AH Martin LJ Olivier M INSIG1 influences obesity-related hypertriglyceridemia in humans J Lipid Res 2010 51 701 708 10.1194/jlr.M001404 19965593 PMC2838707

[B39] Zhang Y Smith EM Baye TM Eckert JV Abraham LJ Moses EK Kissebah AH Martin LJ Olivier M Serotonin (5-HT) receptor 5A sequence variants affect human plasma triglyceride levels Physiol Genomics 2010 42 168 176 10.1152/physiolgenomics.00038.2010 20388841 PMC3032280

[B40] Kissebah AH Sonnenberg GE Myklebust J Goldstein M Broman K James RG Marks JA Krakower GR Jacob HJ Weber J Martin L Blangero J Comuzzie AG Quantitative trait loci on chromosomes 3 and 17 influence phenotypes of the metabolic syndrome Proc Natl Acad Sci USA 2000 97 14478 144783 10.1073/pnas.97.26.14478 11121050 PMC18944

[B41] Svendsen OL Haarbo J Heitmann BL Gotfredsen A Christiansen C Measurement of body fat in elderly subjects by dual-energy x-ray absorptiometry, bioelectrical impedance, and anthropometry Am J Clin Nutr 1991 53 1117 1123 2021122 10.1093/ajcn/53.5.1117

[B42] Peiris AN Hennes MI Evans DJ Wilson CR Lee MB Kissebah AH Relationship of anthropometric measurements of body fat distribution to metabolic profile in premenopausal women Acta Med Scand Suppl 1988 723 179 188 3164966 10.1111/j.0954-6820.1987.tb05942.x

[B43] Bergman RN Toward physiological understanding of glucose tolerance. Minimal-model approach Diabetes 1989 38 1512 1527 10.2337/diabetes.38.12.1512 2684710

[B44] Rainwater DL Moore PH Jr Shelledy WR Dyer TD Slifer SH Characterization of a composite gradient gel for the electrophoretic separation of lipoproteins J Lipid Res 1997 38 1261 1266 9215553

[B45] Dupont NC Wang K Wadhwa PD Culhane JF Nelson EL Validation and comparison of luminex multiplex cytokine analysis kits with ELISA: determinations of a panel of nine cytokines in clinical sample culture supernatants J Reprod Immunol 2005 66 175 191 10.1016/j.jri.2005.03.005 16029895 PMC5738327

[B46] Lee A Nofziger C Dossena S Vanoni S Diasio R Paulmichl M Methylation of the Human Pendrin Promoter Cell Physiol Biochem 2011 28 397 406 10.1159/000335102 22116354

[B47] Göring HH Curran JE Johnson MP Dyer TD Charlesworth J Cole SA Jowett JBM Abraham LJ Rainwater DL Comuzzie AG Mahaney MC Almasy L MacCluer JW Kissebah AH Collier GR Moses EK Blangero J Discovery of expression QTLs using large-scale transcriptional profiling in human lymphocytes Nat Genet 2007 39 10 1208 1216 10.1038/ng2119 17873875

[B48] Richardson B Lu Q Methods for Analyzing the Role of DNA Methylation and Chromatin Structure in Regulating T Lymphocyte Gene Expression Biol Proced 2004 6 189 203 10.1251/bpo89 PMC517978 15448721

[B49] R Development Core Team R: A language and environment for statistical computing. R Foundation for Statistical Computing 2011 Vienna, Austria ISBN 3-900051-07-0, (http://www.R-project.org)

[B50] Almasy L Blangero J Multipoint quantitative-trait linkage analysis in general pedigrees Am J Hum Genet 1998 62 1198 1211 10.1086/301844 9545414 PMC1377101

[B51] Self SG Liang KY Asymptotic properties of maximum likelihood estimators and likelihood ratio tests under nonstandard conditions J Amer Stat Assoc 1987 82 605 610 10.1080/01621459.1987.10478472

[B52] Moskvina V Schmidt KM On multiple-testing correction in genome-wide association studies Genet Epidemiol 2008 32 567 573 10.1002/gepi.20331 18425821

[B53] Qian Q Kuo L Yu TY Rottman JN A Concise Promoter Region of the Hearth Fatty Acid-Binding Protein Gene Dictates Tissue-Appropriate Expression Circ Res 1999 84 276 289 10.1161/01.RES.84.3.276 10024301

[B54] Deng G Chen A Hong J Chae HS Kim YS Methylation of CpG in a Small Region of the hMLH1 Promoter Invariably Correlates with the Absence of Gene Expression Cancer Res 1999 59 2029 2023 10232580

[B55] Zöchbauer-Müller S Fong KM Maitra A Lam S Geradts J Ashfaq R Virmani AK Milchgrub S Gazdar AF Minna JD 5^′^ CpG Island Methylation of the FHIT Gene Is Correlated with Loss of Gene Expression in Lung and Breast Cancer Cancer Res 2001 61 3581 3585 11325823

[B56] Kathiresan S Melander O Guiducci C Surti A Burtt NP Rieder MJ Cooper GM Roos C Voight BF Havulinna AS Wahlstrand B Hedner T Corella D Tai ES Ordovas JM Berglund G Vartiainen E Jousilahti P Hedblad B Taskinen MR Newton-Cheh C Salomaa V Peltonen L Groop L Altshuler DM Orho-Melander M Six new loci associated with blood low-density lipoprotein cholesterol, high-density lipoprotein cholesterol or triglycerides in humans Nat Genet 2008 40 189 197 10.1038/ng.75 18193044 PMC2682493

[B57] Zabaneh D Balding DJ A genome-wide association study of the metabolic syndrome in Indian Asian men PLoS One 2010 8 e11961 20694148 10.1371/journal.pone.0011961 PMC2915922

[B58] Carroll MD Lacher DA Sorlie PD Cleeman JI Gordon DJ Wolz M Grundy SM Johnson CL Trends in serum lipids and lipoproteins of adults, 1960–2002 JAMA 2005 294 14 1773 1781 10.1001/jama.294.14.1773 16219880

[B59] Ebrahim S Beswick A Burke M Davey Smith G Multiple risk factor interventions for primary prevention of coronary heart disease Cochrane Database Syst Rev 2006 4 CD001561 17054138 10.1002/14651858.CD001561.pub2 PMC4160097

[B60] Monami M Lamanna C Desideri CM Mannucci E DPP-4 inhibitors and lipids: systematic review and meta-analysis Adv Ther 2012 29 1 14 25 10.1007/s12325-011-0088-z 22215383

[B61] Nesto RW Beyond low-density lipoprotein: addressing the atherogenic lipid triad in type 2 diabetes mellitus and the metabolic syndrome Am J Cardiovasc Drugs 2005 5 6 379 387 10.2165/00129784-200505060-00005 16259526

[B62] Sharma RK Singh VN Reddy HK Thinking beyond low-density lipoprotein cholesterol: strategies to further reduce cardiovascular risk Vasc Health Risk Manag 2009 5 793 799 19812691 10.2147/vhrm.s5684 PMC2754092

[B63] The ENCODE Project Cosortium An integrated Encyclopedia of DNA Elements in the Human Genome Nature 2012 489 57 74 10.1038/nature11247 22955616 PMC3439153

[B64] Neph S Vierstra J Stergachis AB Reynolds AP Haugen E Vernot B Thurman RE John S Sandstrom R Johnson AK Maurano MT Humbert R Rynes E Wang H Vong S Lee K Bates D Diegel M Roach V Dunn D Neri J Schafer A Hansen RS Kutyavin T Giste E Weaver M Canfield T Sabo P Zhang M Balasundaram G Byron R An expansive human regulatory lexicon encoded in transcription factor footprints Nature 2012 489 83 90 10.1038/nature11212 22955618 PMC3736582

[B65] Thurman RE Rynes E Humbert R Vierstra J Maurano MT Haugen E Sheffield NC Stergachis AB Wang H Vernot B Garg K John S Sandstrom R Bates D Boatman L Canfield TK Diegel M Dunn D Ebersol AK Frum T Giste E Johnson AK Johnson EM Kutyavin T Lajoie B Lee BK Lee K London D Lotakis D Neph S **The accessible chromatin landscape of the human genom**e Nature 2012 489 75 82 10.1038/nature11232 22955617 PMC3721348

[B66] Wang H Maurano MT Qu H Varley KE Gertz J Pauli F Lee K Canfield T Weaver M Sandstrom R Thurman RE Kaul R Myers RM Stamatoyannopoulos JA Widespread plasticity in CTCF occupancy linked to DNA Methylation Genome Res 2012 22 1680 1688 10.1101/gr.136101.111 22955980 PMC3431485

[B67] Kent WJ Sugnet CW Furey TS Roskin KM Pringle TH Zahler AM Haussler D The human genome browser at UCSC Genome Res 2002 12 996 1006 12045153 10.1101/gr.229102 PMC186604

[B68] Rosenbloom KR Dreszer TR Pheasant M Barber GP Meyer LR Pohl A Raney BJ Wang T Hinrichs AS Zweig AS Fujita PA Learned K Rhead B Smith KE Kuhn RM Karolchik D Haussler D Kent WJ ENCODE whole-genome data in the UCSC Genome Browser Nucleic Acids Res 2010 38 D620 D625 10.1093/nar/gkp961 19920125 PMC2808953

[B69] Rosenbloom KR Dreszer TR Long JC Malladi VS Sloan CA Raney BJ Cline MS Karolchik D Barber GP Clawson H Diekhans M Fujita PA Goldman M Gravell RC Harte RA Hinrichs AS Kirkup VM Kuhn RM Learned K Maddren M Meyer LR Pohl A Rhead B Wong MC Zweig AS Haussler D Kent WJ ENCODE whole-genome data in the UCSC Genome Browser: update 2012 Nucleic Acids Res 2011 40 1 6 Database issue 22075998 10.1093/nar/gkr1012 PMC3245183

[B70] Blahnik KR Dou L Echipare L Iyengar S O’Geen H Sanchez E Zhao Y Marra MA Hirst MA Costello JF Korf I Farnham PJ Characterization of the Contradictory Chromatin Signatures at the 3^′^ Exons of Zinc Finger Genes PLoS One 2011 6 e17121 10.1371/journal.pone.0017121 21347206 PMC3039671

[B71] O’Geen H Echipare L Farnham PJ Using ChIP-seq technology to generate high-resolution profiles of histone modifications Methods Mol Biol 2011 791 265 286 10.1007/978-1-61779-316-5_20 21913086 PMC4151291

[B72] O’Geen H Frietze S Farnham PJ Using ChIP-seq Technology to Identify Targets of Zinc Finger Transcription Factors Methods Mol Biol 2010 649 437 455 10.1007/978-1-60761-753-2_27 20680851 PMC4151297

