# Supplementary material for: Fatty acid binding protein 3 (fabp3) is associated with insulin, lipids and cardiovascular phenotypes of the metabolic syndrome through epigenetic modifications in a northern european family population
Source: BMC Med Genomics. 2013 Mar 19;6:9. doi: 10.1186/1755-8794-6-9 (PMC3608249; doi:10.1186/1755-8794-6-9)
Supplement: Additional file 1: Table S1 — PCR Primers for Methylation Detection of the Promoter and First Exon region of FABP3. *Forward Primers contain the T7 promoter tag (5′ AGG AAG AGA G 3′) on the 5′ end of the primer sequence. **Reverse primers contain the T7-promoter tag (5′ CAG TAA TAC GAC TCA CTA TAG GGA GAA GGC T 3′) on the 5′ end of the primer sequence. Primers are complementary to the reverse (-) template strand. [file 1755-8794-6-9-S1.pptx]

## Slide 1
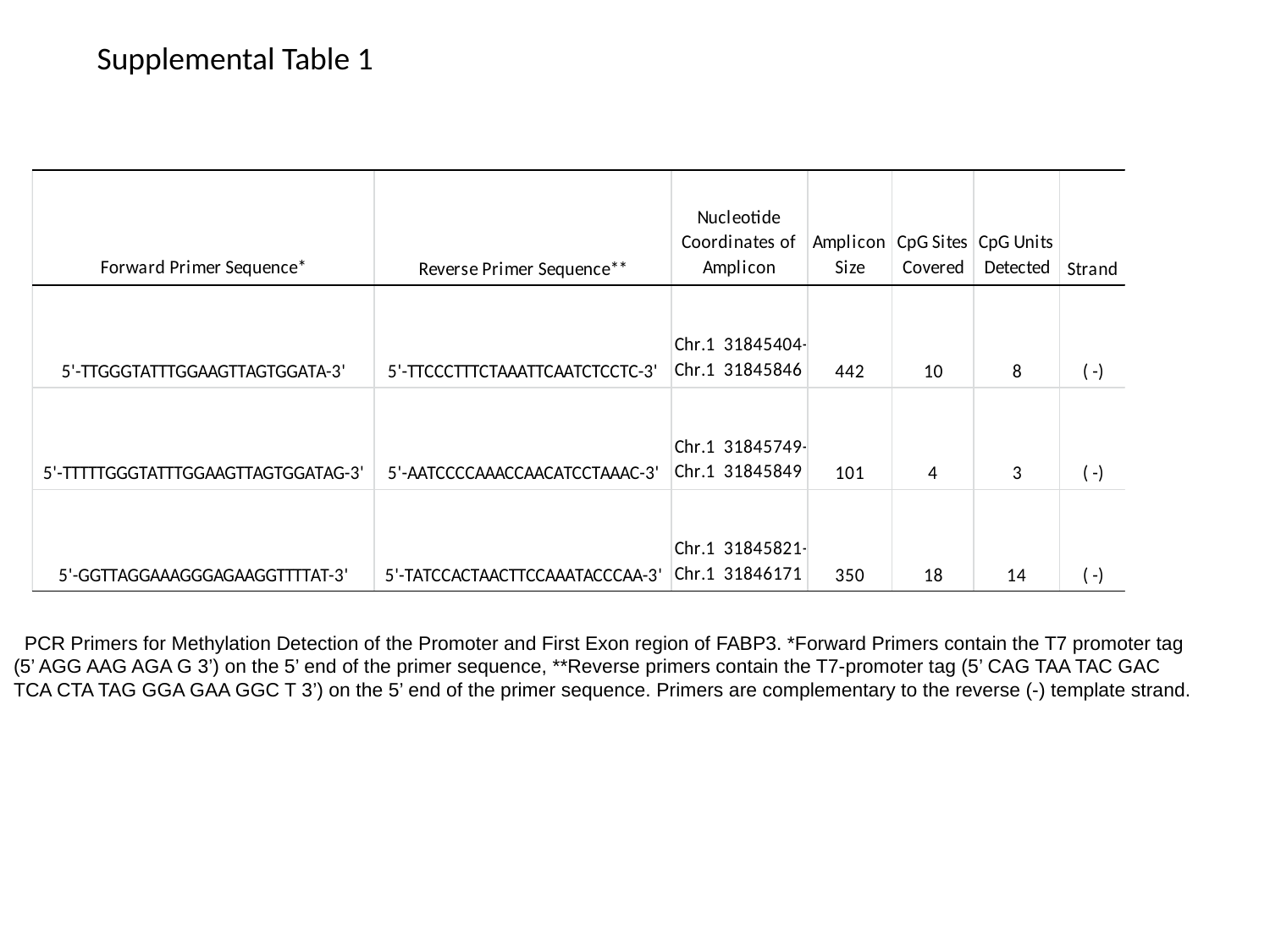

Supplemental Table 1
 PCR Primers for Methylation Detection of the Promoter and First Exon region of FABP3. *Forward Primers contain the T7 promoter tag (5’ AGG AAG AGA G 3’) on the 5’ end of the primer sequence, **Reverse primers contain the T7-promoter tag (5’ CAG TAA TAC GAC TCA CTA TAG GGA GAA GGC T 3’) on the 5’ end of the primer sequence. Primers are complementary to the reverse (-) template strand.
